# One-year follow-up of the effectiveness and mediators of cognitive behavioural therapy among adults with attention-deficit/hyperactivity disorder: secondary outcomes of a randomised controlled trial

**DOI:** 10.1186/s12888-024-05673-8

**Published:** 2024-03-16

**Authors:** Mei-Rong Pan, Min Dong, Shi-Yu Zhang, Lu Liu, Hai-Mei Li, Yu-Feng Wang, Qiu-Jin Qian

**Affiliations:** 1 Peking University Sixth Hospital/ Institute of Mental Health, Beijing, 100191 China; 2 NHC Key Laboratory of Mental Health (Peking University), National Clinical Research Center for Mental Disorders (Peking University Sixth Hospital), Beijing, 100191 China

**Keywords:** Adult, Attention-deficit/hyperactivity disorder, Cognitive behavioural therapy, Maladaptive cognitions, Quality of life, Mediation analysis

## Abstract

**Background:**

The long-term effectiveness of cognitive behavioural therapy (CBT) in medicated attention-deficit/hyperactivity disorder (ADHD) adults with residual symptoms needs to be verified across multiple dimensions, especially with respect to maladaptive cognitions and psychological quality of life (QoL). An exploration of the mechanisms underlying the additive benefits of CBT on QoL in clinical samples may be helpful for a better understanding of the CBT conceptual model and how CBT works in medicated ADHD.

**Methods:**

We conducted a secondary analysis of a randomised controlled trial including 98 medicated ADHD adults with residual symptoms who were randomly allocated to the CBT combined with medication (CBT + M) group or the medication (M)-only group. Outcomes included ADHD-core symptoms (ADHD Rating Scale), depression symptoms (Self-rating Depression Scale), maladaptive cognitions (Automatic Thoughts Questionnaire and Dysfunctional Attitude Scale), and psychological QoL (World Health Organization Quality of Life-Brief Version-psychological domain). Mixed linear models (MLMs) were used to analyse the long-term effectiveness at one-year follow-up, and structural equation modeling (SEM) was performed to explore the potential mechanisms of CBT on psychological QoL.

**Results:**

ADHD patients in the CBT + M group outperformed the M-only group in reduction of ADHD core symptoms (*d* = 0.491), depression symptoms (*d* = 0.570), a trend of reduction of maladaptive cognitions (*d* = 0.387 and 0.395, respectively), and improvement of psychological QoL (*d* = − 0.433). The changes in above dimensions correlated with each other (r = 0.201 ~ 0.636). The influence of CBT on QoL was mediated through the following four pathways: 1) changes in ADHD core symptoms; 2) changes in depressive symptoms; 3) changes in depressive symptoms and then maladaptive cognitions; and 4) changes firstly in depressive symptoms, maladaptive cognitions, and then ADHD core symptoms.

**Conclusions:**

The long-term effectiveness of CBT in medicated ADHD adults with residual symptoms was further confirmed. The CBT conceptual model was verified in clinical samples, which would be helpful for a deeper understanding of how CBT works for a better psychological QoL outcome.

**Trial registration:**

ChiCTR1900021705 (2019-03-05).

## Introduction

Attention-deficit/hyperactivity disorder (ADHD) is a common, chronic neurodevelopmental disorder [1]. Up to 65% of children with ADHD continue to have persistent ADHD symptoms in adulthood [2], and symptoms and associated functional impairments have been shown to fluctuate throughout adulthood in approximately 90% of cases [3]. The prevalence of adult ADHD was approximately 2.5% [4]. Individuals with a diagnosis of ADHD have a greater risk of experiencing depressive symptoms, and difficulties in academic, school, work, marriage, and interpersonal relationships across their lifespan, leading to poor functional outcomes [5, 6].

Psychological quality of life (QoL), which refers to an individual’s subjective and objective evaluations of life functioning and satisfaction, is now more considered as a measure of the long-term outcomes for ADHD [7, 8], rather than merely focusing on changes in the core symptoms [9]. The available evidence still argues for the real benefits of medication in the functional improvement of ADHD adults [10–12] since patients with stable ADHD medication still reported problems with interpersonal and functional impairments [13]. Thus, the importance of nonpharmacological interventions in the treatment of ADHD has been highlighted in addition to pharmacotherapies for better treatment adherence [14] and long-term functional outcomes for adults with ADHD [15].

Cognitive behavioural therapy (CBT) has the most empirical support among nonpharmacotherapies in medicated ADHD adults with residual symptoms [16–19]. Based on the CBT conceptual model of ADHD, QoL impairment might be accounted for by both ADHD core symptoms and the related depressive symptoms since patients received more negative feedback from others caused by core symptom impairments [20]. CBT might improve QoL through two main pathways: the decrease in ADHD core symptoms by acquiring and using compensatory strategies and the release of depressive symptoms via cognition reframing. Besides, the decrease in ADHD core symptoms could also be obtained via the release of depressive symptoms since compensatory strategies could be better used with fewer emotional distress. Studies have demonstrated the effectiveness of CBT in reducing ADHD core symptoms, depression, and improving QoL in adults with ADHD [18, 21, 22], and the important role of depressive symptoms on QoL has also been emphasized [23–25]. However, the potential mechanism based on the CBT conceptual model still needs to be verified in clinical samples, which would benefit from a deeper understanding of how CBT works on the QoL outcome through maladaptive cognitions, depressive symptoms, and ADHD core symptoms, especially on the longer-term impact.

One important way to investigate mechanisms of change is mediation [26], pointing to an intervening variable that may explain the association between the dependent (outcome) and independent (treatment) variables [27]. Most mediating studies have focused on emotional disorders of CBT, and found that the changes in QoL were partially mediated by reductions in symptom changes such as depression [28], sleep [29], and irritable bowel syndrome [30]. However, few studies explored the mediating role in ADHD samples.

Our previous studies confirmed the effectiveness of CBT on ADHD core symptoms, anxiety and depression, maladaptive cognitions, and QoL [22, 31, 32], but long-term effectiveness still needs further confirmation. Additionally, we hope to further explore the mechanism of CBT to determine how CBT works in adult ADHD with residual symptoms, although they have been prescribed with stable medication. Thus, our study aimed to explore (1) the long-term effectiveness of CBT on ADHD core symptoms, maladaptive cognitions, emotional symptoms, and QoL in medicated adult ADHD with residual symptoms, and (2) the possible mechanism of the effect of CBT on QoL through changes in ADHD core symptoms, depressive symptoms, and related maladaptive cognitions using structural equation mediation model. Based on previous studies, the CBT conceptual model, and our research experiences, we hypothesized that (1) CBT could further reduce ADHD core symptoms, depressive symptoms, and related maladaptive cognitions in long-term follow-up, and QoL could be improved; and (2) the change in psychological QoL would be affected indirectly by CBT through ADHD core symptoms, and through depressive symptoms and then related maladaptive cognitions.

## Methods

This study analysed the one-year follow-up data from a published randomised controlled trial (RCT) protocol [33] that compared the effectiveness of CBT between CBT combined with medication (CBT + M) group and medication (M)-only group. The protocol of the original study was registered at Peking University Sixth Hospital, and the registration identification number is ChiCTR1900021705. This study received ethics approval from the ethics committee of Peking University Sixth Hospital, and all participants signed informed consent forms.

### Participants

Participants were outpatients of Peking University Sixth Hospital and individuals recruited from the Internet from March 2019 to November 2019. The outpatients were transferred by psychiatrists in the outpatient clinic. Besides, our research information was also published on the website. Interested participants underwent a screening through recruitment links, and those who initially met the diagnostic criteria through self-assessment screening were then evaluated for eligibility in the outpatient clinic, the same as with those transferred by psychiatrists.

All participants received a diagnostic interview by psychiatric physicians via Conners’ Adult ADHD Diagnostic Interview for DSM-IV (CAADID) [34]. The symptoms during childhood were confirmed based on the recall of the participants as well as the reports of parents or other major caregivers. The key inclusion criteria were as follows:

1. Aged 18–45 years old with a diagnosis of adult ADHD through Conners’ Adult ADHD Diagnostic Interview [34].
2. Stable use of medication (drug fluctuations < 10% for at least 1 month) [35], either methylphenidate hydrochloride controlled-release tablets (Concerta®) or atomoxetine hydrochloride (Strattera®).
3. With residual symptoms since they were scaled with Clinical Global Impression Scale (CGI-S) ≥ 3 (mildly ill or above).

The key exclusion criteria were as follows:

1. Patients with current severe mental disorders, including psychotic disorders, current mania episodes of bipolar disorder, severe depressive episodes with psychotic symptoms or high risk of suicide/self-injury, severe panic disorder, substance abuse, and antisocial personality disorder.
2. Those with a full-scale intelligence quotient (IQ) < 80.
3. Those with suicide risk.
4. Those with unstable physical conditions (such as diabetes, angina pectoris, hypertension, or active hepatitis).
5. Prior or present participation in other psychological therapies.

Patients who attended < 7 times or those who switched to other treatments were considered dropouts from the study.

### Procedures

Participants underwent the assessments at the clinic at three time points, including at baseline, post-treatment, and one-year follow-up. After each time of assessment, the participants would get their multidimensional evaluation report, and the participants of the M-only group were also compensated for an opportunity to participate in group CBT after their one-year follow-up. All participants recruited were under assessment by psychiatrists blinded to treatment assignment, and an independent statistician conducted the randomization. The assessors were postgraduate students of psychiatry who had received unified training to use all the measurement tools, and the consistency was rated.

**Fig. 1:**
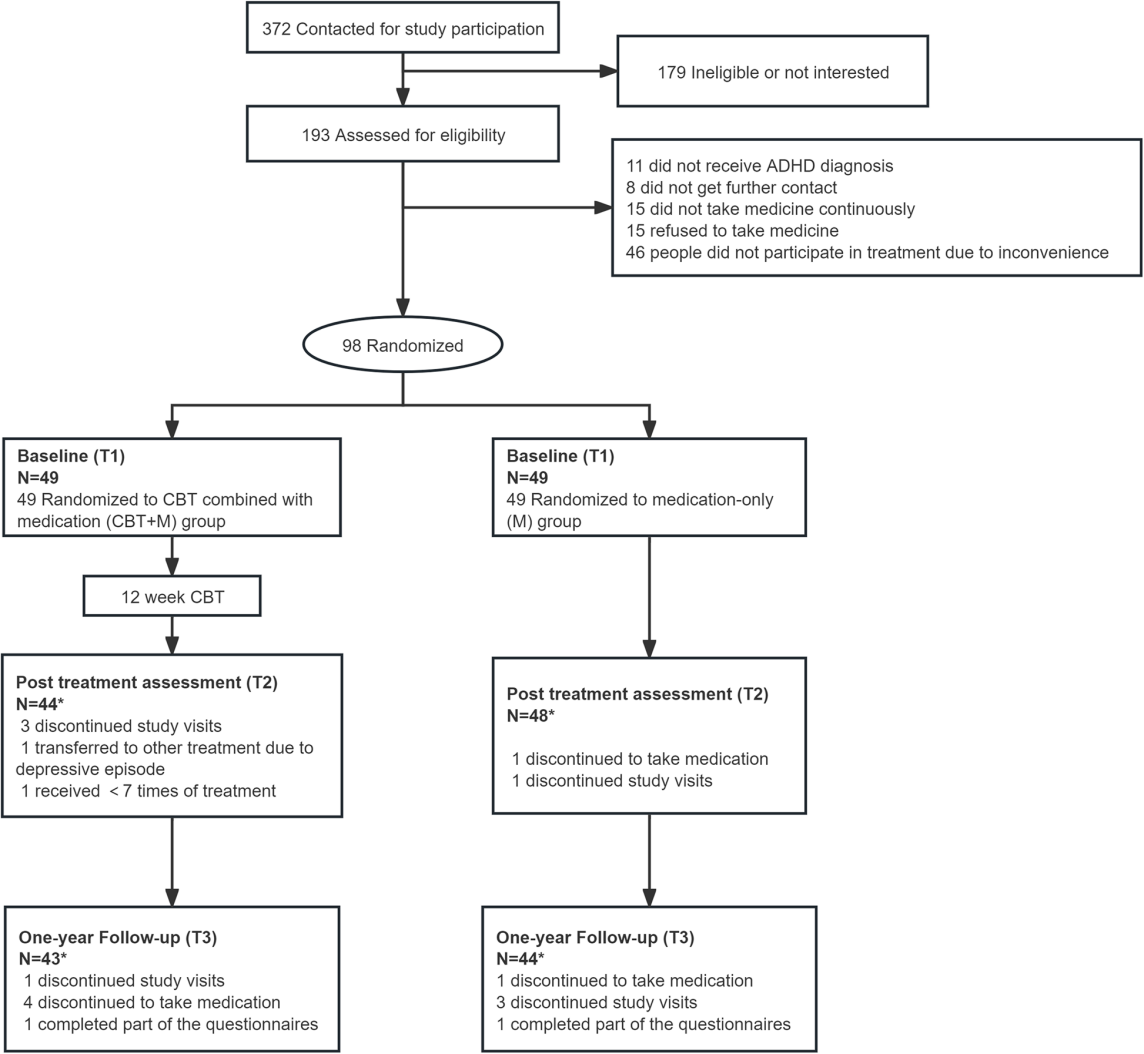
Flow of participants through trial

The flowchart of participants from baseline to one-year follow-up is presented in Fig. 1. A total of 372 subjects interested in the study were contacted, of whom 193 (51.88%) underwent the baseline assessment, and the remaining 98 participants who met the inclusion criteria were randomized to either the CBT + M group (*n* = 49) or the M-only group (n = 49). The CBT + M group engaged in 12 weeks of group CBT, and the M-only group waited and received basic clinical management. One-year follow-up data were obtained from 87.76% (43/49) of the CBT + M group and 89.80% (44/49) of the participants in the M-only group.

### Measures

#### Diagnostic interview and intelligence quotient (IQ) evaluations

Conners’ Adult ADHD Diagnostic Interview for DSM-IV (CAADID) [34] was used to assess ADHD diagnosis, and Structured Clinical Interviews for DSM-IV Axis-I and Axis-II [36] were used to assess comorbid disorders at the baseline assessment. The Wechsler Adult Intelligence Scale-Revised in China (WAIS-RC) [37] was used to estimate the full-scale IQ (FIQ).

#### ADHD core symptoms

The self-reported ADHD Rating Scale (ADHD-RS) [38] was used to evaluate the ADHD core symptoms. The higher the total score, the more severe the core-symptom impairment.

#### Depressive symptoms

The Self-rating Depression Scale (SDS) [39] was used to assess depressive severity. A higher score indicates more severe depressive symptoms.

#### Maladaptive cognitions

The Automatic Thoughts Questionnaire (ATQ) [40] was used to evaluate the frequency of spontaneous negative thoughts, and the Dysfunctional Attitude Scale (DAS) [41] was used to measure individuals’ depressogenic assumptions or beliefs. A higher score indicates more maladaptive cognitions related to depressive symptoms.

#### Quality of life

The World Health Organization Quality of Life-Brief Version (WHOQOL-BREF) [42, 43] was used to evaluate the degree of life satisfaction in four dimensions. We used the psychological domain score to estimate the psychological quality of life (QoL-psychological domain). Higher scores indicate higher levels of QoL.

#### Interventions

The 12-week group CBT program had a detailed introduction in the protocol [33]. The modules on organization and planning, coping with distractibility, restructuring maladaptive cognitions, and dealing with procrastination were mainly discussed. All participants attended CBT organized by the same psychotherapy team who had received systematic training and regular supervision to deliver the program. Each CBT group consisted of 7 to 12 patients, a leader, and a co-leader who cooperated together. The weekly CBT continued for 12 weeks and involved one 120-minute session each week. Each treatment session started with a review of homework and group-based reflection on ADHD and related symptoms, and then new skills learning and discussion were performed, and homework was arranged.

The M-only group received basic clinical management based on their own needs in the outpatient clinic, including medical consultation and nonpharmacological consultation not related to CBT treatment.

Due to the study design, the participants were asked to maintain stable use of medication during follow-up, but best clinical practices were followed, which resulted in some changes. Thus patients were asked to report their use of medication at each time estimation.

#### Statistical analyses

First, independent two-sample t-tests for continuous data and χ2 tests for categorical data were performed to compare the baseline variables. Then, mixed linear models (MLMs) were conducted to analyse the effects between the two groups at the one-year follow-up including dimensions of ADHD core symptoms (ADHD-RS total score), depressive symptoms (SDS score), maladaptive cognitions (ATQ and DAS total scores), and psychological QoL (QoL - psychological domain score). Group, time and group×time interactions were used as fixed effects, and the random effect included the intercept term and slope overtime. If a significant group×time interaction was observed, post hoc pairwise comparisons were then conducted to assess the changes between assessment points within each group. The statistics were based on an intent-to-treat (ITT) analysis, and multiple imputations (data were imputed 5 times) were conducted in the original dataset to address the missing data [44].

The changes in ADHD core symptoms, depressive symptoms, maladaptive cognitions, and QoL at one-year follow-up from baseline were then collected. To explore the mechanism of CBT, Pearson’s correlation was used to assess the correlation among score changes in above dimensions both in the CBT + M and M-only groups. Irrelevant, weak, moderate, and strong correlations (r) were defined as r values of 0–0.09, 0.10–0.30, 0.30–0.50, and 0.50–1.00, respectively. Structural equation mediation model analyses (SEM) were then performed using the R package lavaan [45] with the R software (Version 4.2.2) to test the influence of CBT on changes in psychological QoL via ADHD core symptoms, depressive symptoms, and maladaptive cognitions. The mediation analysis was controlled for baseline dimension indicators (such as age, gender, years of education, FIQ, etc.) if differences between groups were found. Model fit was assessed using the confirmatory fit index (CFI) [46], root mean square error of approximation (RMSEA), and standardized root mean square residual (SRMR) [47]. CFI > 0.95 and RMSEA < 0.06 are viewed as supporting good model fit.

## Results

### Baseline characteristics

No significant differences in the demographic background data were found between the CBT + M and the M-only groups (see Table 1).

**Table 1:** Demographics Clinical Characteristics at baseline

		CBT + M (*n* = 49)	M (*n* = 49)	χ^2^/t value	*p* value
Male(%)		27 (55.1)	31 (63.3)	0.676	0.411
age [mean (SD)]		26.8 (5.7)	24.8 (5.6)	1.816	0.072
FIQ [mean (SD)]		122.8 (9.1)	119.5 (9.8)	1.704	0.092
ADHD subtype (%)					
	Predominantly inattentive	31 (63.3)	28 (57.1)	0.383	0.536
	Combined	18 (36.7)	21 (42.9)		
Year of education [mean (SD)]		16.4 (2.4)	15.7 (2.5)	1.454	0.149
Current Axis I Disorder (%)		21 (42.9)	23 (46.9)	0.165	0.685
≥1 Current Axis I Disorder (%)		7 (14.3)	8 (16.3)	0.079	0.779
Affective Disorder (%)		11 (22.5)	14 (28.6)	0.483	0.487
	Bipolar Disorder	4 (8.16)	4 (8.16)	0.000	1.000
	Major Depressive Disorder (%)	6 (12.24)	9 (18.37)	0.708	0.400
	Dysthymia (%)	1 (2.04)	2 (4.08)	0.344	1.000
Anxiety Disorder (%)		13 (26.5)	10 (20.4)	0.511	0.475
Comorbid Axis I disorders in remission (%)		4 (8.2)	7 (14.3)	0.922	0.337
	Affective Disorder (%)	4 (8.2)	6 (12.2)	0.445	0.505
	Anxiety Disorder (%)	0 (0.0)	1 (6.7)	1.010	1.000
Current comorbid Axis II disorders (%)		27 (55.1)	26 (53.1)	0.041	0.839
≥1 Axis II Disorder (%)		9 (18.4)	11 (22.5)	0.251	0.616
	Cluster A (%)	8 (16.3)	8 (16.3)	0.000	1.000
	Cluster B (%)	4 (8.2)	6 (12.2)	0.445	0.505
	Cluster C (%)	22 (44.9)	19 (38.8)	0.633	0.426
	Other (%)	9 (18.8)	15 (30.6)	1.986	0.159
Medication					
	Methylphenidate (Concerta®)	47 (95.9)	46 (93.9)	1.069	1.000
	Atomoxetine (Strattera®)	2 (4.1)	2 (4.1)		
	Methylphenidate+Atomoxetine	0 (0.0)	1 (2.0)		

*FIQ* full-scaled intelligence quotient; Axis II Cluster A: Paranoid Personality Disorder, Schizoid Personality Disorder, Schizotypal Personality Disorder; Axis II Cluster B: Antisocial personality disorder, Borderline personality disorder, Schizophrenic personality disorder, Narcissistic personality disorder; Axis II Cluster C: Avoidance personality disorder, Dependent personality disorder, Obsessive-Compulsive Personality Disorder; Axis II Other: Personality Change Due to Another Medical Condition, Other Specified Personality Disorder, Unspecified Personality Disorder. CBT + M: the CBT combined with medication group; M: the medication-only group

### Treatment adherence

During the 12-week CBT, the participants attended a mean of 10.76 ± 1.38 sessions. The dropout rate did not differ significantly between the CBT + M group vs the M-only group after 12 weeks of CBT (CBT + M: 10.20%, M: 4.08%, χ2 = 1.385, *p* = 0.436) and at one-year follow-up (CBT + M: 12.24%, M: 10.20%, χ2 = 0.102, *p* = 0.749) (Fig. 1).

In the CBT + M group, one patient changed the use of medication from Strattera® to a combination of Strattera® and Concerta®. One patient stopped taking the medication after the 12-week treatment and 4 of them discontinued the medication at the one-year follow-up. In the M-only group, one participant stopped using the medication after the 12-week treatment and one of them discontinued the medication at the one-year follow-up. All patients completed the follow-up assessments whether or not they took medication regularly. Analyses that both included and excluded the follow-up data from these participants were conducted, and no differences in results were found, so the final results contained the whole data.

### Long-term effectiveness

Table 2 displayed the means, SEs, and differential treatment effects of the CBT + M group versus the M-only group on ADHD core symptoms, depressive symptoms, maladaptive cognitions, and psychological QoL from baseline to one-year follow-up.

**Table 2:** Means, SEs, and differential treatment effects (Cohen’s *d*) of the CBT + M group versus the M-only group from baseline to one-year follow-up

	Baseline [mean (SE)]		One-year follow-up [mean (SE)]		Cohen’s d (95%CI)
	CBT + M	M	CBT + M	M	
ADHD-RS	25.8 (1.5)	23.5 (1.5)	18.3 (1.1)***	21.2 (1.1)	0.491 (0.088, 0.892)
SDS	41.7 (1.4)	41.6 (1.4)	37.5 (1.5)***	42.2 (1.5)	0.570 (0.164, 0.973)
WHOQoL-Psychological domain	46.2 (2.5)	46.8 (2.5)	53.1 (2.7)***	46.9 (2.7)	−0.433 (−0.827, −0.022)
ATQ	67.6 (3.3)	68.7 (3.3)	60.0 (3.8)*	69.3 (3.8)	0.387 (− 0.014, 0.786)
DAS	150.0 (4.7)	146.0 (4.7)	137.0 (4.5)***	144.0 (4.5)	0.395 (− 0.006, 0.794)

*ADHD-RS* ADHD-Rating Scale: *SDS* Self-Rating Depression Scale: *WHOQOL-BREF* World Health Organization Quality of Life-Brief Version: *CBT + M* the CBT combined with medication group: *M* the medication-only group

* *p* < 0.05; **: *p* < 0.01; ***: *p* < 0.001

First, patients in the CBT + M group had a significantly greater decrease in self-reported ADHD-RS scores at the one-year follow-up than those in the M-only group (group×time interaction: *d* = 0.491, *95% CI* = [0.088, 0.892]). Post hoc pairwise comparisons within each group indicated that the self-reported ADHD-RS total score decreased significantly (*p <* 0.001) in the CBT + M group but remained stable in the M-only group (*p* = 0.144).

Second, the CBT + M group outperformed the M-only group in the decrease of SDS score (group×time interaction: *d* = 0.570, *95% CI* = [0.164, 0.973]) and improvement of WHOQOL-BREF psychological domain score (group×time interaction: *d* = − 0.433, *95% CI* = [− 0.827, − 0.022]). Post hoc pairwise comparisons within each group indicated that the SDS scores (*p* <  0.001) decreased significantly, and the WHOQoL-psychological domain score (*p* = 0.012) increased significantly in the CBT + M group. Patients in the CBT + M group also achieved significant improvements in ATQ (t = 2.507, *p* = 0.014) and DAS (t = 3.263, *p* = 0.002) scores from baseline at one-year follow-up, although the group×time interactions were not statistically significant (*p* = 0.058 and 0.054, respectively). Patients in the M-only group maintained stable scores (*p* > 0.05) in the above dimensions.

### Correlations between changes in ADHD core symptoms, depressive symptoms, maladaptive cognitions, and psychological QoL

Pearson’s correlation analyses were used to evaluate the relationships in adults with ADHD after controlling for sex, age, and FIQ. Positive correlations between changes in ADHD-RS and SDS, ATQ, and DAS (r = 0.201 ~ 0.472, *p* <  0.01) were found, and the correlations were small to moderate. Changes in SDS score were positively correlated with changes in ATQ and DAS score (r = 0.365 ~ 0.541, *p* <  0.001), and the WHOQOL-BREF-psychological domain score (r = 0.500 in the CBT + M group and 0.499 in the M-only group, *p* < 0.001). The above correlations presented similar both in the CBT + M group and in the M-only group. The correlations between changes in ADHD-RS total score and WHOQOL-BREF psychological domain score in the M-only group were strong (r = 0.537) and moderate in the CBT + M group (r = 0.317) (Table 3).

**Table 3:** Correlations among the changes in ADHD core symptoms, depression symptoms, maladaptive cognitions, and QoL at one-year follow-up

	CBT + M Group (n = 49)				M-only Group (n = 49)			
	ADHD-RS	SDS	ATQ	DAS	ADHD-RS	SDS	ATQ	DAS
SDS	0.261***				0.276***			
ATQ	0.229***	0.541***			0.472***	0.527***		
DAS	0.201**	0.365***	0.636***		0.265***	0.383***	0.518***	
WHOQOL-BREF Psychological domain	0.317***	0.500***	0.513***	0.351***	0.537***	0.499***	0.542***	0.339***

*ADHD-RS* ADHD-Rating Scale: *SDS* Self-Rating Depression Scale: *ATQ* Automatic Thoughts Questionnaire: *DAS* Dysfunctional Attitudes Scales: *WHOQOL-BREF* World Health Organization Quality of Life-Brief Version: *CBT + M* the CBT combined with medication group: *M* the medication-only group

**p < 0.01; ***: *p* < 0.001

### Mediation analyses

All variables of ADHD core symptoms, depressive symptoms, and maladaptive cognitions were selected. The independent variable was CBT intervention, and the dependent variable included change in QoL-psychological domain score, mediators included changes in ADHD-RS total score, SDS, and maladaptive cognitions (ATQ and DAS).

**Fig. 2:**
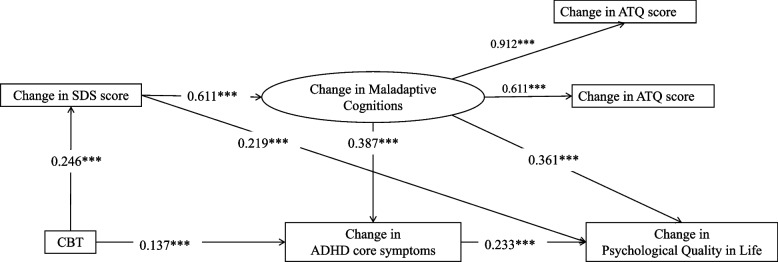
The influence of CBT on psychological QoL via changes in ADHD core symptoms, depression, and maladaptive cognitions at one-year follow-up

The structural model for CBT on QoL and maladaptive cognitions showed a good fit (χ2 (df = 5.000) = 7.999, *p* = 0.156, CFI = 0.996, RMSEA = 0.035, SRMR = 0.025). The model showed that CBT would influence changes in WHOQOL-psychological domain score through the following pathways: 1) the change in ADHD-RS total score (β = 0.032, 95% *CI* = [0.399, 1.854], *p* = 0.004); 2) the change in SDS score (β = 0.054, 95% *CI* = [0.782, 2.795], *p* < 0.001); and 3) the change in SDS score and then the change in maladaptive cognitions (β = 0.054, 95% *CI* = [0.966, 3.052], *p* < 0.001). Besides, CBT would also influence the change in ADHD-RS total score through the change in SDS score and then the change in maladaptive cognitions, thereby affecting the change in WHOQOL-psychological domain score (β = 0.014, 95% *CI* = [0.223, 0.749], *p* < 0.001) (Table 4 and Fig. 2).

**Table 4:** The influence of CBT on psychological QoL via changes in ADHD core symptoms, depression, and maladaptive cognitions at one-year follow-up

Effects:	Standardized Coefficients (95% *CI*)	*P* value	Unstandardized Coefficients	S.E.
Total effect	0.153 (3.453, 6.892)	< 0.001***	5.087	−0.864
Pathway				
CBT → Change in ADHD-RS → Change in Psychological QoL	0.032 (0.399, 1.854)	0.004**	1.062	−0.373
CBT → Change in SDS → Change in Psychological QoL	0.054 (0.782, 2.795)	< 0.001***	1.781	−0.496
CBT → Change in SDS → Change in Maladaptive Cognitions→Change in Psychological QoL	0.054 (0.966, 3.052)	< 0.001***	1.796	−0.519
CBT → Change in SDS → Change in Maladaptive Cognitions→Change in ADHD-RS → Change in Psychological QoL	0.014 (0.223, 0.749)	0.001**	0.448	−0.140

*ADHD-RS* ADHD-Rating Scale, *SDS* Self-Rating Depression Scale: Maladaptive Cognitions, ATQ and DAS *ATQ* Automatic Thoughts Questionnaire, *DAS* Dysfunctional Attitudes Scales, *QoL* Quality of Life (World Health Organization Quality of Life-Brief Version-Psychological domain score)

**p < 0.01; ***: *p* < 0.001

## Discussion

The study had the following findings. First, ADHD patients in the CBT + M group outperformed the M-only group in the reduction of ADHD core symptoms, depressive symptoms, a trend of reduction of maladaptive cognitions, and improvement of the psychological domain of QoL. Second, the changes in ADHD core symptoms, depressive symptoms, maladaptive cognitions, and psychological QoL correlated with each other. Besides, the influence of CBT on QoL was mediated through the following four pathways: 1) changes in ADHD core symptoms; 2) changes in depressive symptoms; 3) changes in depressive symptoms and then maladaptive cognitions; and 4) changes firstly in depressive symptoms, maladaptive cognitions, and then ADHD core symptoms.

### Long-term effectiveness

First, we found that adults in the CBT + M group had a greater reduction in ADHD core symptoms when compared with the M-only group, consistent with previous studies compared with the treatment as usual at follow-up 12 weeks after the end of treatment [18, 21, 48] and relaxation training [16] and clinical management [49] at the 12-month follow-up. Our study further confirmed the effectiveness of CBT on medicated patients in the long-term follow-up. The superiority of CBT on ADHD core symptoms mainly results in regular and repeated practice of compensatory strategies, such as organization and planning skills in daily life, which could be accumulated and bring long-term benefits, consistent with our assumption in previous studies [22, 31]. One study [50] found the effectiveness of CBT on core symptoms might be masked if patients performed good responses to medication, as studies indicated the benefits of medication on ADHD core symptoms [51, 52]. Thus, a combination of CBT can be an effective supplementary treatment for core symptoms impairment in addition to stable medication.

Additionally, the CBT + M group showed a larger improvement in self-reported depression symptoms than the M-only group during the long-term follow-up, in line with the studies of Safren [16] but contrasted with the findings of Philipsen [49] and Corbisiero [50]. Similar to ADHD core symptoms, the emotional symptom reductions in the CBT + M group would be masked since medication also benefits patients’ emotional dysregulation and emotional symptoms related to ADHD [51, 53]. Previous studies also found equal effectiveness of CBT in depressive symptoms when combined with medication or not [32, 54, 55], leading to the hypothesis that CBT might be more effective in depressive symptoms.

This study indicated the effectiveness of CBT in reducing maladaptive cognitions in patients with ADHD, in line with those patients with depression [56], insomnia [57], and agoraphobia [58]. The ATQ was used to assess the frequency of negative automatic thoughts [40], and the DAS was developed to assess the beliefs or attitudes that underlie the characteristic cognitive content, especially with depression [41]. Patients with ADHD were found to have shown elevated scores of maladaptive cognitions, which correlated with emotional symptoms [59]. CBT helps ADHD patients improve their cognitive flexibility and reframe their maladaptive cognitions, and the long-term effectiveness would also be obtained through the benefits in the improvement of depressive symptoms.

Our study emphasized the effectiveness of CBT treatment on psychological QoL, in line with a previous RCT at the 3-month follow-up [19, 21, 48] with moderate to large effect sizes. The WHOQOL-psychological domain emphasizes an individual’s positive feelings, personal beliefs, and self-esteem [60], which could be improved by CBT from positive reinforcement, intimacy, interpersonal learning, social support [61], and self-efficacy accumulation supported by the therapist [62].

### Correlations between changes in ADHD core symptoms, depressive symptoms, maladaptive cognitions, and psychological QoL

The results found positive relationships between the changes in depressive symptoms, maladaptive cognitions, and QoL, in line with studies on depression [56]. The positive relationship between core symptoms and QoL changes was in line with the RCT study of ADHD adults [19]. Besides, we found that the correlation between changes in ADHD core symptoms and QoL was strong in the M-only group but moderate in the CBT + M group, indicating that other factors would also be correlated with life quality change, such as emotional changes caused by CBT [63]. A previous study found that medicated adults with ADHD still have emotional distress and maladaptive cognitions [64], and CBT helps patients improve negative emotions and comorbid symptoms, which would be beneficial to better QoL improvement.

### Mediation analyses

We first explored the effect of CBT on QoL in adults with ADHD in the long-term follow-up and found that the changes in ADHD core symptoms, depression, and maladaptive cognitions are all direct or indirect mediators of the influence of CBT on QoL.

First, the influence of CBT on QoL through ADHD core symptoms emphasized the importance of core symptom reduction in QoL improvement. Besides, the mediating role of ADHD core symptoms also exists through depressive symptoms and then maladaptive cognition changes. CBT helps ADHD patients develop problem-solving strategies and compensatory skills that target core symptoms, and the reduction of core symptom impairment would be beneficial to functional outcomes [7]. Besides, changes in negative emotions and then related maladaptive cognitions are also considered positive factors of core symptoms and QoL prognosis, since the compensatory strategies and skills learning could be better obtained with fewer depressive symptoms.

Second, we found that the changes in depressive symptoms fully mediated the relationship between CBT and QoL. The decrease in depression was related to increases in QoL [65], and changes in mental health-related QoL over the course of treatment were accounted for by changes in depression [63]. A similar mediating role has also been found in emotional distress related to insomnia [29] and physical diseases [66, 67]. Both group cohesion and CBT-related skills training can improve emotional symptoms [68–70]. Additionally, the accumulation of positive feedback and support from others and interpersonal learning in groups can bring long-term benefits, such as improvements in depressive symptoms, and then benefit QoL improvement when extended to daily life [71, 72]. The mediating role of maladaptive cognitions was also observed via the change in depressive symptoms. Dysfunctional attitudes are proposed to be a consequence of depressive symptom reduction [73, 74], and the reduction of depression would also improve the related dysfunctional attitudes and negative automatic thoughts, which could then bring a better QoL outcome.

### Clinical and research implications

Our study explored the effectiveness of CBT in adults with ADHD in the long-term follow-up in multiple dimensions, and first explored the potential mechanism of CBT based on the CBT conceptual model in clinical samples. These findings indicate the possibility and necessity for clinicians to pay more attention to the residual symptoms of medicated ADHD, especially on their QoL, and the potential psychological mechanisms from the CBT perspective. Besides, our findings also highlight the important role in reducing emotional distress when working with ADHD due to the significant functional impacts via poor experiences accumulation and emotional distress [62]. Also, preventive interventions for emotional problems and functional impairments in the ADHD population are essential across the lifespan, which have already been explored preliminarily in adolescents [75] and would be further considered and implemented in adulthood in future studies.

### Limitations of study

There were several limitations of our study. First, we focused on the effectiveness of CBT on QoL and their mediating factors including improvement of ADHD core symptoms, depression, and maladaptive cognitions, since participants involved in our study still struggled with emotional and QoL distress. The role of compensatory strategies and targeted, skills-based interventions and their relationship with maladaptive cognitions and emotions need to be explored in future studies. Second, an additional CBT-only group without medication could be included in future RCT studies to explore the role and mechanism of medication, CBT, and the combination of these treatments. Also, a broader range of participants with years of education and intellectual level is needed for future studies to identify the optimal combination, integration, and timing of known efficacious CBT interventions in different populations.

## Conclusion

Our study further confirmed the long-term effectiveness of CBT on ADHD core symptoms, depressive symptoms, maladaptive cognitions, and psychological QoL in medicated adults with ADHD with residual symptoms. The CBT conceptual model in clinical samples was verified, indicating that the influence of CBT on QoL was mediated through changes in ADHD core symptoms, changes in depressive symptoms, and changes in depressive symptoms and then maladaptive cognitions. The mediating role of ADHD core symptoms also exists indirectly through the changes in emotional symptoms and then cognitive patterns. Our study provides a scientific basis for efficacious CBT interventions for ADHD patients, especially for those with residual symptoms. The potential mechanism was explored for a deeper understanding of how CBT works for a better QoL outcome.

## Data Availability

The raw data analysed during the current study will be made available from the corresponding author on reasonable request.

## Abbreviations

ADHD: Attention-Deficit/Hyperactivity-Disorder
ADHD-RS: ADHD Rating Scale
CBT: Cognitive Behavioural Therapy
QoL: Quality of Life
RCT: Randomised Controlled Trial
ITT: Intention-to-treat
MLM: Mixed linear model
SEM: Structural Equation Modeling
